# 2,3-Diamino­pyridinium (2*E*,4*E*)-hexa-2,4-dienoate

**DOI:** 10.1107/S1600536810032617

**Published:** 2010-08-21

**Authors:** Madhukar Hemamalini, Hoong-Kun Fun

**Affiliations:** a X-ray Crystallography Unit, School of Physics, Universiti Sains Malaysia, 11800 USM, Penang, Malaysia

## Abstract

In the title salt, C_5_H_8_N_3_^+^·C_6_H_7_O_2_^−^, the pyridine N atom of the 2,3-diamino­pyridine mol­ecule is protonated. The 2,3-diamino­pyridinium cation is essentially planar, with a maximum deviation of 0.068 (2) Å for one of the amino N atoms. The sorbate anion adopts an extended conformation. In the crystal structure, the protonated N atom and one of the two amino-group H atoms are hydrogen bonded to the sorbate anion through a pair of N—H⋯O hydrogen bonds, forming an *R*_2_^2^(8) ring motif. The ion pairs are further connected *via* inter­molecular N—H⋯O and C—H⋯O hydrogen bonds, forming two-dimensional networks parallel to (100).

## Related literature

For details of non-covalent inter­actions, see: Desiraju (1995 ▶); Moulton & Zaworotko (2001 ▶); Biradha & Fujita (2002 ▶). For applications of sorbic acid, see: Martindale (1996 ▶); Richards (1972 ▶). For related structures, see: Cox (1994 ▶); Thanigaimani *et al.* (2007 ▶); Raj *et al.* (2003 ▶). For hydrogen-bond motifs, see: Bernstein *et al.* (1995 ▶). For standard bond-length data, see: Allen *et al.* (1987 ▶).
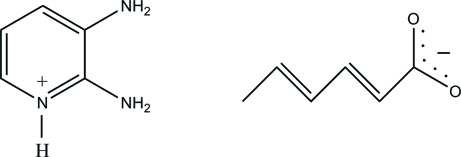


## Experimental

### 

#### Crystal data

C_5_H_8_N_3_^+^·C_6_H_7_O_2_^−^
*M* *_r_* = 221.26
Monoclinic, 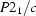

*a* = 9.0440 (3) Å
*b* = 10.6964 (3) Å
*c* = 12.4632 (4) Å
β = 94.947 (2)°
*V* = 1201.18 (6) Å^3^
*Z* = 4
Mo *K*α radiation
μ = 0.09 mm^−1^
*T* = 296 K
0.15 × 0.15 × 0.09 mm

#### Data collection

Bruker SMART APEXII CCD area-detector diffractometer
Absorption correction: multi-scan (*SADABS*; Bruker, 2009 ▶) *T*_min_ = 0.987, *T*_max_ = 0.993
8260 measured reflections
2736 independent reflections
1794 reflections with *I* > 2σ(*I*)
*R*_int_ = 0.053

#### Refinement

*R*[*F* ^2^ > 2σ(*F* ^2^)] = 0.049
*wR*(*F* ^2^) = 0.111
*S* = 1.02
2736 reflections
205 parameters
All H-atom parameters refined
Δρ_max_ = 0.22 e Å^−3^
Δρ_min_ = −0.22 e Å^−3^

### 

Data collection: *APEX2* (Bruker, 2009 ▶); cell refinement: *SAINT* (Bruker, 2009 ▶); data reduction: *SAINT*; program(s) used to solve structure: *SHELXTL* (Sheldrick, 2008 ▶); program(s) used to refine structure: *SHELXTL*; molecular graphics: *SHELXTL*; software used to prepare material for publication: *SHELXTL* and *PLATON* (Spek, 2009 ▶).

## Supplementary Material

Crystal structure: contains datablocks global, I. DOI: 10.1107/S1600536810032617/lh5118sup1.cif

Structure factors: contains datablocks I. DOI: 10.1107/S1600536810032617/lh5118Isup2.hkl

Additional supplementary materials:  crystallographic information; 3D view; checkCIF report

## Figures and Tables

**Table 1:** Hydrogen-bond geometry (Å, °)

*D*—H⋯*A*	*D*—H	H⋯*A*	*D*⋯*A*	*D*—H⋯*A*
N1—H1*N*1⋯O2	1.00 (2)	1.66 (2)	2.6591 (19)	174.7 (18)
N2—H1*N*2⋯O2	0.86 (2)	2.05 (2)	2.9065 (18)	173.1 (17)
N2—H2*N*2⋯O1	0.94 (2)	1.90 (2)	2.8432 (19)	176.2 (18)
N3—H1*N*3⋯O2	0.89 (2)	2.04 (2)	2.930 (2)	174 (2)
N3—H2*N*3⋯O1^i^	0.91 (2)	2.11 (2)	2.913 (2)	146.9 (16)
C10—H10⋯O1^ii^	0.983 (19)	2.531 (18)	3.330 (2)	138.2 (14)

Symmetry codes: (i) 
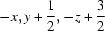
; (ii) 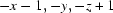.

**Fig. 1.**
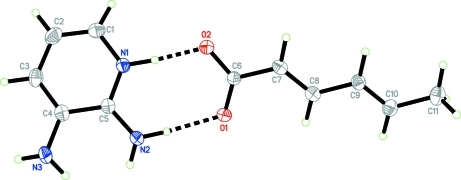
The asymmetric unit of the title compound. Displacement ellipsoids are drawn at the 50% probability level. Hydrogen bonds are shown as dashed lines.

**Fig. 2.**
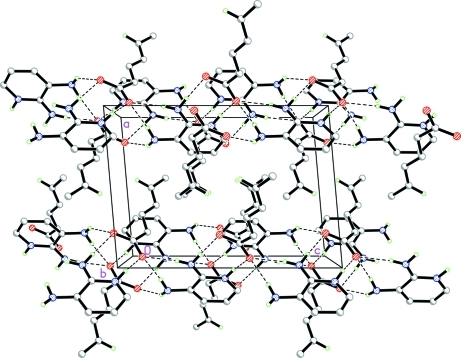
The crystal packing of (I) showing hydrogen bonds as dashed lines. H atoms not involved in the intermolecular interactions have been omitted for clarity.

## supplementary crystallographic information

### Comment

Non-covalent interactions, such as hydrogen bonds, π···π staking and the C—H···π interactions, play important roles in determining the conformation of molecules, crystal packing and molecular assembly in an organized supramolecular structure (Desiraju, 1995; Moulton & Zaworotko, 2001; Biradha & Fujita, 2002). Sorbic acid (2,4-hexadienoic acid) exhibits antibacterial and antifungal properties (Martindale, 1996) and has been used to prevent spoilage of syrup by moulds (Richards, 1972).  The crystal structure of sorbic acid (Cox, 1994), 2,4-diamino-6-phenyl-1,3,5-triazine-sorbic acid (Thanigaimani *et al.*, 2007) and trimethoprim sorbate (Raj *et al.*, 2003) have been reported in the literature.  Since our aim is to study some interesting hydrogen-bonding interactions, the crystal structure of the title compound is presented herein.

The asymmetric unit of the title compound, (Fig 1), contains one 2,3-diaminopyridinium cation and one sorbate anion. The bond lengths (Allen *et al.*, 1987) and angles are normal. The 2,3-diaminopyridinium cation is planar with a maximum deviation of 0.068 (2) Å for atom N3. Protonation  of atom N1 has resulted in a slight increase in the magnitude of angle C1—N1—C5 [122.86 (16)°]. The sorbic acid moiety is in the *EE* configuration. The extended conformation of the sorbic acid can be described by the torsion angles C6-C7-C8-C9 = -173.77 (16)°, C7-C8-C9-C10 = 176.15 (18)°, C8-C9-C10-C11 = -176.92 (18)° and O2-C6-C7-C8 = 171.14 (16)°. This conformation is similar to that found in the trimethoprim sorbate dihydrate (Raj *et al.*, 2003).

In the crystal structure, (Fig. 2), the protonated N1 atom and the 2-amino group N atom (N2) is hydrogen-bonded to the carboxylate oxygen atoms (O1 and O2) via a pair of N1—H1N1···O2 and N2—H2N2···O1 hydrogen bonds forming a ring motif *R*_2_^2^(8) (Bernstein *et al.*, 1995). The ion pairs are further connected via  N2—H1N2···O2^i^; N3—H1N3···O2^i^; N3—H2N3···O1^ii^ and C10—H10···O1^iii^ hydrogen bonds (see Table 1 for symmetry codes) forming two-dimensional networks parallel to (100).

### Experimental

A hot methanol solution (20 ml) of 2,3-diaminopyridine (59 mg, Aldrich) and sorbic acid (56 mg, Merck) were mixed and warmed over a heating magnetic stirrer hotplate for a few minutes. The resulting solution was allowed to cool slowly at room temperature and crystals of the title compound appeared after a few days.

### Refinement

All H atoms were located in a difference Fourier map and refined freely [C–H = 0.94 (2)–1.04 (2) Å and N–H = 0.86 (2)–1.00 (2) Å].

### Crystal data

**Table:**

C_5_H_8_N_3_^+^·C_6_H_7_O_2_^−^	*F*(000) = 472
*M**_r_* = 221.26	*D*_x_ = 1.224 Mg m^−^^3^
Monoclinic, *P*2_1_/*c*	Mo *K*α radiation, λ = 0.71073 Å
Hall symbol: -P 2ybc	Cell parameters from 1444 reflections
*a* = 9.0440 (3) Å	θ = 3.3–28.9°
*b* = 10.6964 (3) Å	µ = 0.09 mm^−^^1^
*c* = 12.4632 (4) Å	*T* = 296 K
β = 94.947 (2)°	Block, red
*V* = 1201.18 (6) Å^3^	0.15 × 0.15 × 0.09 mm
*Z* = 4	

### Data collection

**Table:**

Bruker SMART APEXII CCD area-detector diffractometer	2736 independent reflections
Radiation source: fine-focus sealed tube	1794 reflections with *I* > 2σ(*I*)
graphite	*R*_int_ = 0.053
φ and ω scans	θ_max_ = 27.5°, θ_min_ = 2.5°
Absorption correction: multi-scan (*SADABS*; Bruker, 2009)	*h* = −11→8
*T*_min_ = 0.987, *T*_max_ = 0.993	*k* = −13→11
8260 measured reflections	*l* = −16→16

### Refinement

**Table:**

Refinement on *F*^2^	Primary atom site location: structure-invariant direct methods
Least-squares matrix: full	Secondary atom site location: difference Fourier map
*R*[*F*^2^ > 2σ(*F*^2^)] = 0.049	Hydrogen site location: inferred from neighbouring sites
*wR*(*F*^2^) = 0.111	All H-atom parameters refined
*S* = 1.02	*w* = 1/[σ^2^(*F*_o_^2^) + (0.0417*P*)^2^ + 0.1735*P*] where *P* = (*F*_o_^2^ + 2*F*_c_^2^)/3
2736 reflections	(Δ/σ)_max_ < 0.001
205 parameters	Δρ_max_ = 0.22 e Å^−^^3^
0 restraints	Δρ_min_ = −0.22 e Å^−^^3^

### Special details

**Table:**

Geometry. All s.u.'s (except the s.u. in the dihedral angle between two l.s. planes) are estimated using the full covariance matrix. The cell s.u.'s are taken into account individually in the estimation of s.u.'s in distances, angles and torsion angles; correlations between s.u.'s in cell parameters are only used when they are defined by crystal symmetry. An approximate (isotropic) treatment of cell s.u.'s is used for estimating s.u.'s involving l.s. planes.
Refinement. Refinement of F^2^ against ALL reflections. The weighted R-factor wR and goodness of fit S are based on F^2^, conventional R-factors R are based on F, with F set to zero for negative F^2^. The threshold expression of F^2^ > 2σ(F^2^) is used only for calculating R-factors(gt) etc. and is not relevant to the choice of reflections for refinement. R-factors based on F^2^ are statistically about twice as large as those based on F, and R- factors based on ALL data will be even larger.

### Fractional atomic coordinates and isotropic or equivalent isotropic displacement parameters (Å^2^)

**Table:**

	*x*	*y*	*z*	*U*_iso_*/*U*_eq_	
O1	−0.19667 (13)	0.14636 (12)	0.53361 (8)	0.0233 (3)	
O2	−0.04642 (13)	0.18916 (12)	0.40526 (8)	0.0241 (3)	
C6	−0.14500 (18)	0.12200 (17)	0.44625 (11)	0.0180 (4)	
H7	−0.148 (2)	−0.0069 (18)	0.3182 (13)	0.026 (5)*	
H8	−0.364 (2)	−0.0391 (18)	0.4703 (14)	0.030 (5)*	
H9	−0.317 (2)	−0.1811 (18)	0.2736 (14)	0.028 (5)*	
H10	−0.545 (2)	−0.2085 (19)	0.4233 (15)	0.037 (6)*	
H11A	−0.672 (3)	−0.307 (2)	0.2669 (16)	0.050 (6)*	
H11B	−0.556 (2)	−0.407 (2)	0.3260 (14)	0.031 (5)*	
H11C	−0.507 (2)	−0.3389 (19)	0.2230 (15)	0.034 (5)*	
C7	−0.1975 (2)	0.01064 (18)	0.38280 (12)	0.0224 (4)	
C8	−0.3120 (2)	−0.06038 (18)	0.40571 (12)	0.0209 (4)	
C9	−0.3730 (2)	−0.16160 (17)	0.33880 (13)	0.0219 (4)	
C10	−0.4924 (2)	−0.22749 (18)	0.35940 (14)	0.0252 (4)	
C11	−0.5606 (2)	−0.3280 (2)	0.28818 (17)	0.0309 (5)	
H2N3	0.224 (2)	0.516 (2)	0.8425 (15)	0.035 (6)*	
N1	0.09874 (16)	0.36086 (15)	0.52745 (10)	0.0212 (4)	
N2	0.00087 (17)	0.28443 (16)	0.67865 (12)	0.0236 (4)	
N3	0.1886 (2)	0.44680 (19)	0.80698 (11)	0.0286 (4)	
C1	0.1867 (2)	0.43933 (19)	0.47441 (14)	0.0278 (5)	
C2	0.2698 (2)	0.5265 (2)	0.52935 (15)	0.0324 (5)	
C3	0.2682 (2)	0.53273 (19)	0.64186 (14)	0.0276 (5)	
C4	0.18421 (19)	0.45140 (18)	0.69625 (12)	0.0222 (4)	
C5	0.09207 (18)	0.36429 (17)	0.63493 (12)	0.0193 (4)	
H1	0.178 (2)	0.4288 (18)	0.3983 (14)	0.027 (5)*	
H2	0.324 (2)	0.586 (2)	0.4933 (15)	0.038 (6)*	
H3	0.326 (2)	0.599 (2)	0.6825 (14)	0.032 (5)*	
H1N1	0.039 (2)	0.297 (2)	0.4839 (15)	0.037 (6)*	
H1N2	−0.021 (2)	0.291 (2)	0.7442 (17)	0.038 (6)*	
H2N2	−0.065 (2)	0.236 (2)	0.6330 (16)	0.036 (6)*	
H1N3	0.113 (3)	0.407 (2)	0.8330 (16)	0.049 (7)*	

### Atomic displacement parameters (Å^2^)

**Table:**

	*U*^11^	*U*^22^	*U*^33^	*U*^12^	*U*^13^	*U*^23^
O1	0.0270 (7)	0.0241 (8)	0.0191 (5)	−0.0026 (6)	0.0040 (5)	−0.0010 (5)
O2	0.0274 (7)	0.0258 (8)	0.0193 (5)	−0.0058 (6)	0.0023 (5)	0.0002 (5)
C6	0.0193 (9)	0.0172 (11)	0.0170 (7)	0.0011 (8)	−0.0020 (6)	0.0033 (7)
C7	0.0238 (10)	0.0241 (12)	0.0190 (7)	0.0022 (9)	0.0005 (7)	−0.0010 (7)
C8	0.0206 (9)	0.0205 (12)	0.0211 (7)	0.0050 (8)	−0.0006 (7)	0.0010 (7)
C9	0.0237 (10)	0.0191 (12)	0.0225 (8)	0.0016 (8)	0.0002 (7)	−0.0009 (7)
C10	0.0243 (10)	0.0218 (12)	0.0295 (9)	0.0013 (9)	0.0023 (7)	0.0001 (8)
C11	0.0272 (11)	0.0276 (14)	0.0373 (10)	−0.0041 (10)	−0.0007 (9)	−0.0009 (10)
N1	0.0237 (8)	0.0201 (9)	0.0197 (6)	−0.0022 (7)	0.0010 (6)	−0.0009 (6)
N2	0.0252 (9)	0.0272 (11)	0.0187 (7)	−0.0052 (8)	0.0034 (6)	−0.0046 (7)
N3	0.0309 (10)	0.0300 (11)	0.0246 (7)	−0.0088 (8)	0.0012 (7)	−0.0094 (7)
C1	0.0320 (11)	0.0280 (13)	0.0235 (8)	−0.0025 (9)	0.0033 (8)	0.0042 (8)
C2	0.0346 (12)	0.0280 (14)	0.0351 (9)	−0.0075 (10)	0.0053 (9)	0.0068 (9)
C3	0.0293 (11)	0.0183 (12)	0.0346 (9)	−0.0039 (9)	−0.0001 (8)	−0.0032 (8)
C4	0.0207 (9)	0.0206 (11)	0.0249 (8)	0.0029 (8)	0.0003 (7)	−0.0029 (8)
C5	0.0185 (9)	0.0167 (11)	0.0223 (7)	0.0030 (8)	0.0006 (6)	−0.0012 (7)

### Geometric parameters (Å, °)

**Table:**

O1—C6	1.2488 (18)	N1—C1	1.366 (2)
O2—C6	1.285 (2)	N1—H1N1	1.00 (2)
C6—C7	1.485 (2)	N2—C5	1.336 (2)
C7—C8	1.335 (2)	N2—H1N2	0.86 (2)
C7—H7	0.974 (17)	N2—H2N2	0.95 (2)
C8—C9	1.446 (2)	N3—C4	1.378 (2)
C8—H8	0.993 (18)	N3—H2N3	0.91 (2)
C9—C10	1.333 (3)	N3—H1N3	0.89 (3)
C9—H9	1.017 (18)	C1—C2	1.347 (3)
C10—C11	1.493 (3)	C1—H1	0.952 (16)
C10—H10	0.982 (19)	C2—C3	1.405 (2)
C11—H11A	1.04 (2)	C2—H2	0.94 (2)
C11—H11B	0.97 (2)	C3—C4	1.372 (3)
C11—H11C	0.99 (2)	C3—H3	0.99 (2)
N1—C5	1.3466 (19)	C4—C5	1.427 (2)
			
O1—C6—O2	123.69 (16)	C1—N1—H1N1	117.8 (11)
O1—C6—C7	120.34 (15)	C5—N2—H1N2	122.4 (14)
O2—C6—C7	115.97 (14)	C5—N2—H2N2	119.2 (12)
C8—C7—C6	124.12 (15)	H1N2—N2—H2N2	115.5 (18)
C8—C7—H7	119.4 (11)	C4—N3—H2N3	116.1 (12)
C6—C7—H7	116.4 (11)	C4—N3—H1N3	115.1 (14)
C7—C8—C9	124.52 (16)	H2N3—N3—H1N3	117.3 (19)
C7—C8—H8	118.2 (11)	C2—C1—N1	120.15 (16)
C9—C8—H8	117.1 (11)	C2—C1—H1	125.5 (12)
C10—C9—C8	124.02 (16)	N1—C1—H1	114.3 (12)
C10—C9—H9	121.0 (11)	C1—C2—C3	119.12 (19)
C8—C9—H9	114.9 (11)	C1—C2—H2	121.0 (12)
C9—C10—C11	124.47 (17)	C3—C2—H2	119.8 (12)
C9—C10—H10	120.1 (12)	C4—C3—C2	121.12 (19)
C11—C10—H10	115.3 (12)	C4—C3—H3	119.4 (11)
C10—C11—H11A	109.4 (13)	C2—C3—H3	119.5 (11)
C10—C11—H11B	110.0 (11)	C3—C4—N3	123.23 (17)
H11A—C11—H11B	108.2 (17)	C3—C4—C5	118.16 (15)
C10—C11—H11C	111.7 (11)	N3—C4—C5	118.57 (17)
H11A—C11—H11C	110.2 (16)	N2—C5—N1	118.07 (16)
H11B—C11—H11C	107.3 (16)	N2—C5—C4	123.47 (15)
C5—N1—C1	122.86 (16)	N1—C5—C4	118.45 (16)
C5—N1—H1N1	119.3 (11)		
			
O1—C6—C7—C8	8.5 (3)	C2—C3—C4—N3	174.29 (19)
O2—C6—C7—C8	−171.14 (16)	C2—C3—C4—C5	−3.3 (3)
C6—C7—C8—C9	173.77 (16)	C1—N1—C5—N2	179.13 (17)
C7—C8—C9—C10	−176.15 (18)	C1—N1—C5—C4	−1.8 (3)
C8—C9—C10—C11	176.92 (18)	C3—C4—C5—N2	−177.03 (18)
C5—N1—C1—C2	−1.2 (3)	N3—C4—C5—N2	5.3 (3)
N1—C1—C2—C3	2.0 (3)	C3—C4—C5—N1	4.0 (3)
C1—C2—C3—C4	0.3 (3)	N3—C4—C5—N1	−173.69 (16)

### Hydrogen-bond geometry (Å, °)

**Table:**

*D*—H···*A*	*D*—H	H···*A*	*D*···*A*	*D*—H···*A*
N1—H1N1···O2	1.00 (2)	1.66 (2)	2.6591 (19)	174.7 (18)
N2—H1N2···O2^i^	0.86 (2)	2.05 (2)	2.9065 (18)	173.1 (17)
N2—H2N2···O1	0.94 (2)	1.90 (2)	2.8432 (19)	176.2 (18)
N3—H1N3···O2^i^	0.89 (2)	2.04 (2)	2.930 (2)	174 (2)
N3—H2N3···O1^ii^	0.91 (2)	2.11 (2)	2.913 (2)	146.9 (16)
C10—H10···O1^iii^	0.983 (19)	2.531 (18)	3.330 (2)	138.2 (14)

Symmetry codes: (i) ; (ii) −*x*, *y*+1/2, −*z*+3/2; (iii) −*x*−1, −*y*, −*z*+1.
